# Interplay of swine acute diarrhoea syndrome coronavirus and the host intrinsic and innate immunity

**DOI:** 10.1186/s13567-024-01436-1

**Published:** 2025-01-09

**Authors:** Fei Zhao, Xiao Cong, Xiaobo Huang, Yi Zheng, Qin Zhao, Yiping Wen, Rui Wu, Senyan Du, Sanjie Cao, Feng Cong, Yiping Wang

**Affiliations:** 1 Department of Preventive Veterinary Medicine, Research Center for Swine Diseases, College of Veterinary Medicine, Sichuan Agricultural University, Chengdu, 611130 China; 2 Guangdong Laboratory Animals Monitoring Institute, Guangzhou, 510663 Guangdong China; 3 Key Laboratory of Agricultural Bioinformatics of Ministry of Education, Sichuan Agricultural University, Chengdu, 611130 China

**Keywords:** Swine acute diarrhoea syndrome coronavirus, SADS-CoV, intrinsic immunity, innate immunity, immune evasion

## Abstract

Swine acute diarrhoea syndrome coronavirus (SADS-CoV), a novel HKU2-related coronavirus of bat origin, is a newly emerged swine enteropathogenic coronavirus that causes severe diarrhoea in piglets. SADS-CoV has a broad cell tropism with the capability to infect a wide variety of cells from human and diverse animals, which implicates its ability to hold high risks of cross-species transmission. The intracellular antiviral immunity, comprised of the intrinsic and innate immunity, represents the first line of host defence against viral infection prior to the onset of adaptive immunity. To date, there are no vaccines and drugs approved to prevent or treat SADS-CoV infection. Understanding of the mutual relationship between SADS-CoV infection and host immunity is crucial for the development of novel vaccines and drugs against SADS-CoV. Here, we review recent advancements in our understanding of the interplay between SADS-CoV infection and the host intrinsic and innate immunity. The extensive and in-depth investigation on their interactive relationship will contribute to the identification of new targets for developing intervention strategies to control SADS-CoV infection.

## Introduction

Coronaviruses are a large group of enveloped positive-sense single-stranded RNA viruses that belong to the subfamily *Coronavirinae*, family *Coronaviridae*, and order *Nidovirales* [1]. Based on the genetic properties, the *Coronavirinae* family is categorized into four genera: *Alphacoronavirus*, *Betacoronavirus*, *Gammacoronavirus*, and *Deltacoronavirus* [1]. Coronavirus infection can cause mild to severe respiratory and digestive tract diseases in a wide range of wild and domesticated birds and mammals, including humans, posing a huge threat to animal and human health [1]. Currently, the known swine enteropathogenic coronaviruses that cause diarrhea in pigs include transmissible gastroenteritis virus (TGEV), porcine epidemic diarrhea virus (PEDV), swine acute diarrhea syndrome coronavirus (SADS-CoV), and porcine deltacoronavirus (PDCoV) [2]. Among them, TGEV, PEDV, and SADS-CoV belong to the *Alphacoronavirus* genus, while PDCoV is a member in the *Deltacoronavirus* genus [3].

SADS-CoV, also known as porcine enteric alphacoronavirus (PEAV) and swine enteric alphacoronavirus (SeACoV), is a newly emerged swine enteric coronavirus that was first discovered in 2017 in the Guangdong Province, China [4–6]. SADS-CoV is a novel HKU2-related coronavirus that spills over from bat to cause severe diseases in domestic animals [4–6]. SADS-CoV causes severe vomiting, watery diarrhoea, dehydration, and high mortality rates in newborn piglets, leading to enormous economic losses in the pig industry [4–6]. Fortunately, in addition to Guangdong [4–8], SADS-CoV has been reported to cause sporadic outbreaks only in four other Provinces in China so far, including Fujian, Jiangxi, Guangxi, and Henan [9–12].

The RNA genome of SADS-CoV is approximately 27 kilobases long, which contains a 5′-untranslated region (UTR), open reading frame 1a (ORF1a), ORF1b, spike protein (S), ORF3/NS3a, envelope protein (E), membrane protein (M), nucleocapsid protein (N), NS7a, NS7b, and 3′-UTR [12, 13]. Within the 5′ two thirds of the genome, ORF1a and ORF1b encode polyprotein 1a (pp1a) and pp1b, respectively, which are cleaved by two virus-encoded proteases, papain-like protease 2 (PLP2) and 3 chymotrypsin-like protease (3CLPro), to generate 16 nonstructural proteins (NSP1-16) involved in viral replication and transcription [12, 13]. PLP2 and 3CLPro are encoded by *NSP3* and *NSP5* genes, respectively. The remaining 3′ one third of the genome expresses four structural proteins (S, E, M and N) and three accessory proteins (ORF3/NS3a, NS7a, and NS7b) [12, 13]. The S protein consists of the S1 and S2 subunits, which mediate receptor binding and membrane fusion, respectively, to promote viral entry [14–16]. The E and M proteins are main components of viral envelop essential for viral assembly and release, and N protein typically participates in virion packaging by binding to viral genomic RNA [12, 13]. However, the functions of the three accessory proteins are largely unknown.

SADS‐CoV is capable of infecting a large variety of cell lines from humans and animals, including swine, chickens, monkeys, cats, dogs, mice, rats, hamsters, mink, and bats [17–20]. Although SADS-CoV has not been reported to infect humans, its broad host tropism implicates that SADS‐CoV holds a high risk of cross-species transmission and poses a potential threat to public health [21]. To gain entry into the cells, SADS‐CoV is first attached to heparan sulfate and sialic acid on the target cell surfaces [16], then utilizes the S1 subunit of S protein to bind cellular receptors and the S2 subunit to trigger membrane fusion. While N-linked glycosylation of host cells plays an important role in SADS-CoV attachment [22], the specific N-linked glycoproteins mediating this process remain unknown. SADS‐CoV entry requires cleavage of S protein by diverse host proteases, including furin, cathepsin L, cathepsin B, transmembrane protease serine 2 (TMPRSS2), TMPRSS4, and TMPRSS13 [14–16, 22, 23]. Furthermore, bile acids, a common type of microbial metabolites, were identified to enhance SADS-CoV entry in porcine intestinal enteroids through caveolae-mediated endocytosis [24]. However, SADS‐CoV does not use the known coronavirus functional receptors, including angiotensin converting enzyme 2 (ACE2), dipeptidyl peptidase 4 (DPP4), and aminopeptidase N (APN), for cellular entry [6, 17, 18]. Though the precise mechanisms of SADS-CoV entry remain elusive, the recent generation of recombinant vesicular stomatitis virus with its G protein replaced by SADS-CoV S protein and green fluorescent protein-labelled recombinant SADS-CoV will provide a valuable platform for accelerating the identification of the functional receptors for SADS-CoV entry [15, 16].

To date, no vaccines and drugs are commercially available to defend SADS-CoV infection. Understanding the interplay between SADS-CoV and host antiviral immunity is critical for expediting the research and development of vaccines and drugs against SADS-CoV infection. Therefore, this review summarizes recent advancements in our understanding of the interplay of SADS-CoV and the host intrinsic and innate immunity, providing new insight into the complex relationship between SADS-CoV and intracellular antiviral immunity.

## Intrinsic versus innate immunity

**Figure 1:**
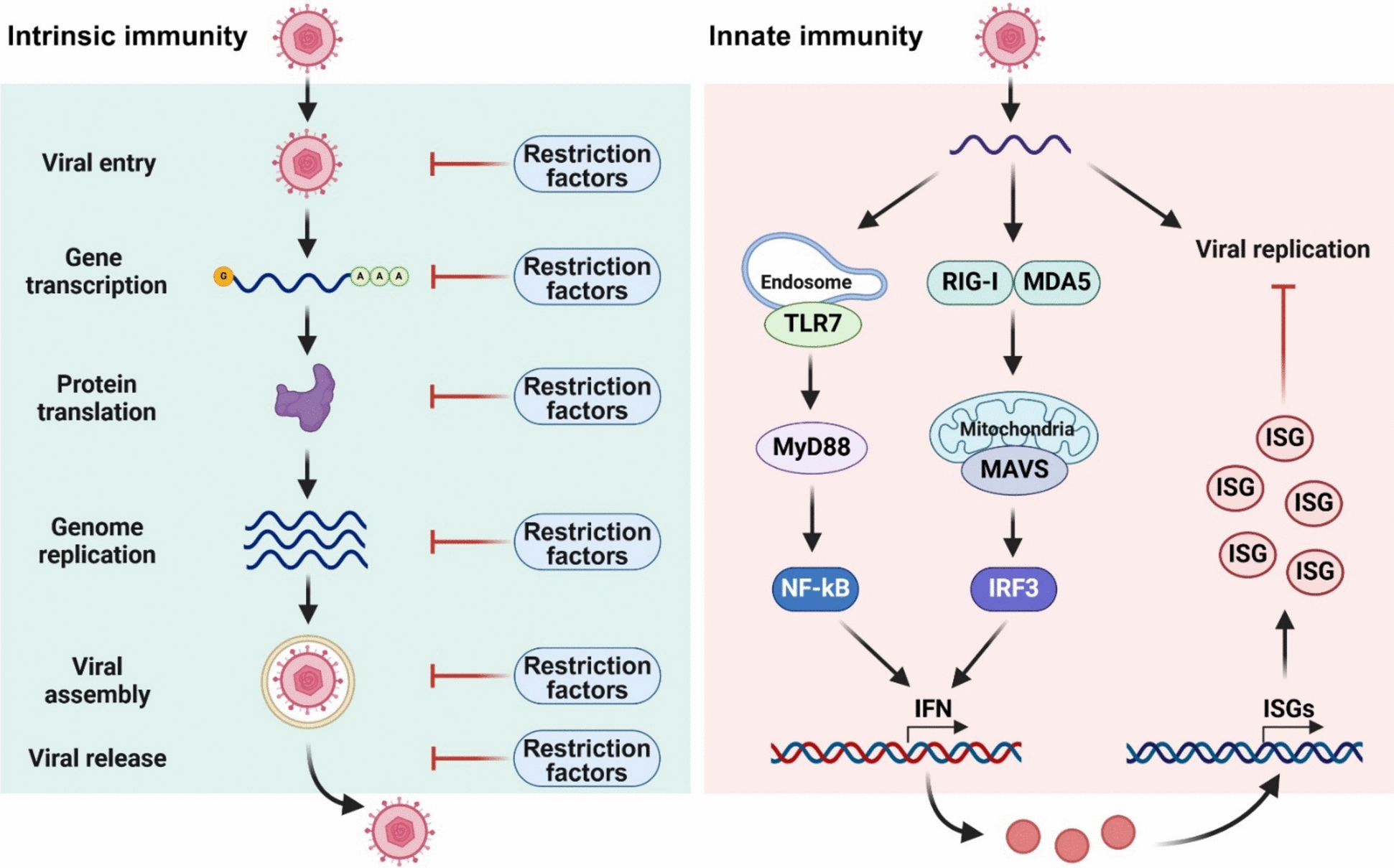
**Intrinsic versus innate immunity**. During Intrinsic immunity, the constitutively expressed host restriction factors exert antiviral activities in different stages of viral life cycle. In contrast, invading viral DNA or RNA are recognized by specific host PRR (such as TLR7, RIG-I, and MDA5) during innate immunity that signal to induce IFN secretion, which further triggers the expression of numerous ISG to restrict viral replication. Figure created with BioRender.

Upon virus infection, intracellular antiviral immunity serves as the first line of host defense against invading viruses before the onset of adaptive immunity. Based on the requirements for the interferon (IFN) system, host intracellular antiviral immunity can be divided into two major arms: (1) intrinsic immunity (also known as cell-intrinsic or cell-autonomous immunity) and (2) innate immunity [25–30]. Intrinsic immunity refers to an IFN-independent antiviral response conferred by constitutively expressed cellular proteins that are known as intrinsic host restriction factors or intrinsic host antiviral factors (Figure 1). While these factors are typically preexistent in certain cell types, they can be further induced by viral infection. The host restriction factors restrain viral replication immediately and directly after infection, often prior to the beginning of the IFN response. However, these factors can also be upregulated by IFN, which can remarkably enhance their antiviral activities to better inhibit viral replication. In the past decades, numerous cellular proteins have been identified as the host restriction factors, including interferon-induced transmembrane (IFITM) proteins [31–35], cholesterol 25-hydroxylase (CH25H) [36–38], interferon-inducible IFI16 protein [39–41], optineurin (OPTN) [42], and transmembrane protein 53 (TMEM53) [43], that are able to target different stages of viral life cycle for viral inhibition.

In contrast, innate immunity represents an IFN-dependent antiviral response mediated by cellular receptors that are known as pattern recognition receptors (PRR) (Figure 1). The well characterized mammalian PRR include toll-like receptors (TLR), retinoic acid-inducible gene I (RIG-I)-like receptors (RLR), the nucleotide-binding oligomerization domain (NOD)-like receptors (NLR), and the cytosolic DNA sensor stimulator of interferon genes (STING) [44]. Following the recognition of pathogen-associated molecular patterns (PAMP) of viruses (such as viral nucleic acids) by host PRR, the associated cellular signalling pathways are activated. Taking RLR signalling pathway for example, the sensors RIG-I and melanoma differentiation-associated protein 5 (MDA5) are activated by viral RNA, then interact with the caspase activation and recruitment domain (CARD) on mitochondrial antiviral signalling protein (MAVS), which acts as the critical adaptor protein to mediate downstream signal transduction [45, 46]. MAVS relays the signal to TANK-binding kinase 1 (TBK1) and inhibitor of nuclear factor kappa B (IκB) kinase-ε (IKKε) through tumour necrosis factor (TNF) receptor-activated factor 3 (TRAF3), which causes activation of the transcription factors (TF) including interferon regulatory factor (IRF3), IRF7, and nuclear factor kappa B (NF-κB) [45, 46]. Activated TF are then transported into the nucleus where they trigger transcription of the genes encoding IFN and proinflammatory cytokines [45, 46].

The IFN are classified into three different families: type I IFN (IFNα, IFNβ, IFNε, IFNτ, IFNκ, IFNω, IFNδ, and IFNζ), type II IFN (IFNγ), and type III IFN (IFNλ1, INFλ2, IFNλ3, and IFNλ4) [26, 47]. All three types of IFN have an inherent ability to induce the expression of IFN-stimulated genes (ISG) in an autocrine and paracrine manner, which can create an antiviral cellular environment to restrict viral replication [26, 47]. The type I and type II IFN receptors, the heterodimeric IFNα receptor 1/2 (IFNAR1/IFNAR2) and IFNγ receptor 1/2 (IFNGR1/IFNGR2) complexes, respectively, are ubiquitously expressed almost in all cell types, while the expression of type III IFN receptors, the heterodimeric IFNλ receptor 1/IL10 receptor 2 (IFNLR1/IL10R2) complex, is restricted in the epithelial cells of mucosal surfaces [48]. After binding to their cognate receptors, type I and type III IFN activate the Janus kinase (JAK)-signal transducer and activator of transcription (STAT) signalling pathway to generate the heterotrimeric transcription factor complex interferon-stimulated gene factor 3 (ISGF3), which is composed of phosphorylated STAT1/STAT2 heterodimers and IRF9 [47, 49]. Likewise, type II IFN also activate the JAK-STAT pathway, leading to the formation of so-called IFNγ-activated factor (GAF), which consists of phosphorylated STAT1 homodimers [47]. Activated ISGF3 and GAF are subsequently transported to the nucleus where they induce the expression of hundreds of ISG by binding their promoter elements, IFN-stimulated response elements (ISRE) and gamma-activated sequences (GAS), respectively [47].

## Intrinsic immunity to SADS-CoV

### CH25H

CH25H is a member in the redox enzyme family that is primarily located in the endoplasmic reticulum (ER) and Golgi apparatus. CH25H catalyses the oxidation of cholesterol to 25-hydroxycholesterol (25HC), which is a type of endogenous hydroxysterol that acts in cholesterol homeostasis [26, 50]. When the levels of cellular cholesterol are increased, 25HC reduces its accumulation by suppressing the activities of sterol regulatory element-binding protein (SREBP), which induces the expression of genes associated with cholesterol biosynthesis [51]. Furthermore, 25HC promotes cholesterol trafficking into the ER [26]. Therefore, CH25H and its enzymatic product 25HC are generally thought to play essential roles in maintaining cholesterol homeostasis. However, 25HC can also participate in multiple important cellular processes, including lipid metabolism, antivirus process, inflammatory response, and cell survival [52].

**Figure 2:**
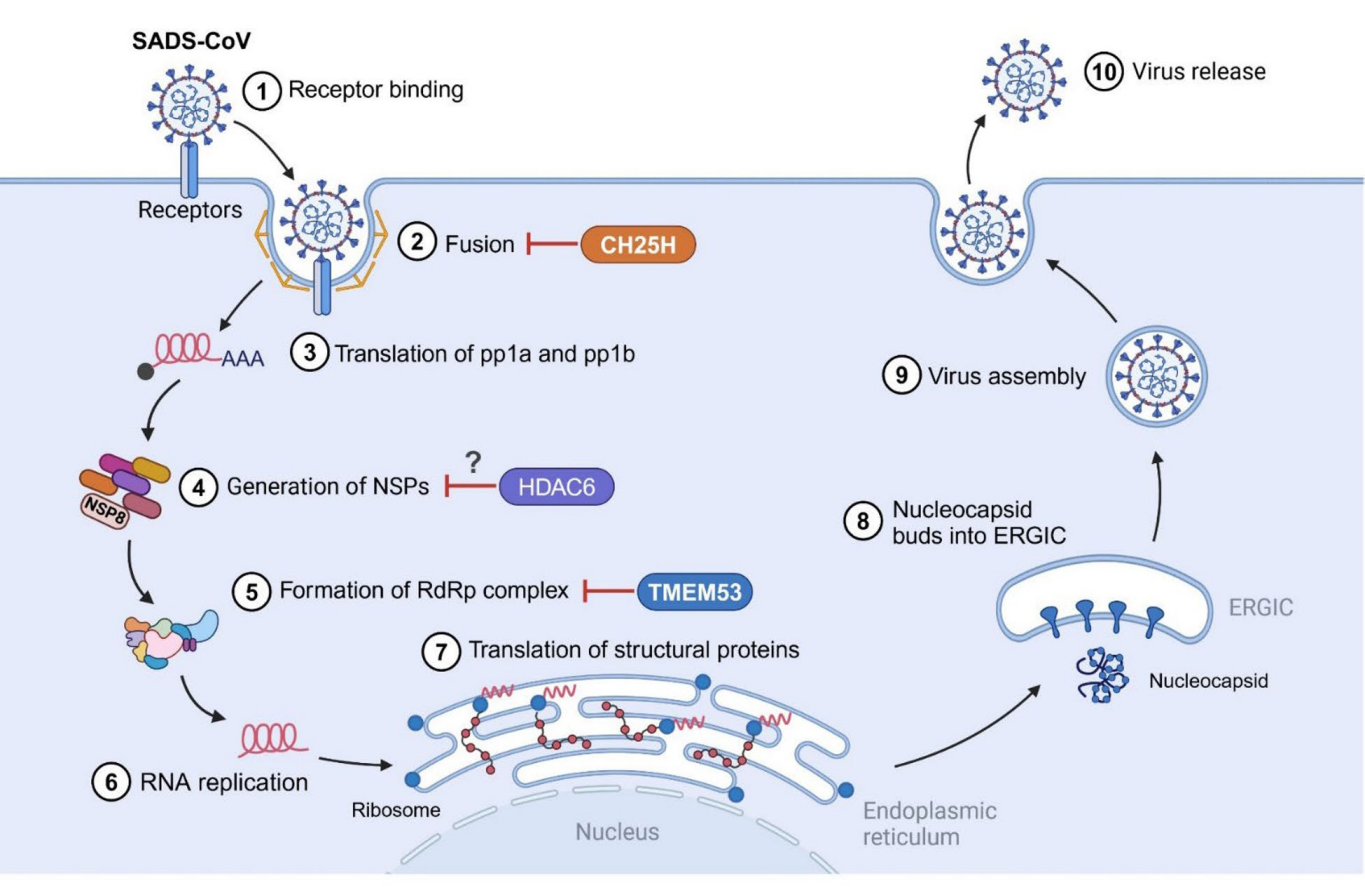
**Intrinsic host restriction factors against SADS-CoV**. The identified host restriction factors CH25H, TMEM53, and HDAC6 target different stages of SADS-CoV life cycle to inhibit viral replication: CH25H blocks S protein-mediated membrane fusion, TMEM53 disrupts the formation of viral RdRp complex, and HDAC6 might induce NSP8 degradation. Figure created with BioRender.

CH25H is an IFN-induced enzyme that is generally upregulated in response to viral infection. It has recently been demonstrated that CH25H has a broad antiviral activity against numerous viruses through various mechanisms, such as inhibition of virus-cell fusion and regulation of membrane cholesterol [26]. SADS-CoV infection upregulates CH25H expression not only in porcine intestinal epithelial cells IPI-2I and Vero E6 cells in vitro, but also in the ileal tissues of piglets in vivo [38]. Treatment of IPI-2I cells with IFNα markedly increases CH25H expression, indicating that porcine CH25H is an ISG [38]. Consistent with the findings for other swine enteric coronaviruses PEDV, TGEV, and PDCoV [53, 54], CH25H and 25HC also inhibit SADS-CoV entry into the cells [38]. Mechanistically, both CH25H and 25HC block S protein-mediated membrane fusion, thus suppressing SADS-CoV replication (Figure 2).

### TMEM53

TMEM53 is a nuclear envelope transmembrane protein that is localized in the outer membrane of the nucleus [55, 56]. The biological function of TMEM53 is largely unknown. The findings from limited studies reveal that TMEM53 regulates cell cycle in a tissue- and cell type-dependent manner and its deficiency is associated with sclerosing bone disorder [57, 58]. The antiviral activity of TMEM53 was first identified through a large-scale human cDNA library screening for potential host restriction factors against SADS-CoV [43]. Mechanistic study unveils that TMEM53 interacts with SADS-CoV NSP12 protein to disrupt NSP8-NSP12 interaction, which interferes with the formation of viral RNA-dependent RNA polymerase (RdRp) complex (Figure 2), thus inhibiting RdRp activity and viral RNA synthesis [43]. Notably, TMEM53 displays broad antiviral activities against multiple closely related bat HKU2-related coronaviruses with zoonotic potential [43]. These important findings suggest that TMEM53 may serve as a promising therapeutic target against SADS-CoV and HKU2-related coronavirus infection [43]. Because TMEM53 is a newly identified host restriction factor, the information on its antiviral activity and the underlying mechanisms is still very rare. It is therefore warranted to determine whether TMEM53 restrains the replication of other viruses, especially other swine enteric coronaviruses.

### HDAC6

Histone deacetylase 6 (HDAC6) is a unique cytoplasmic deacetylase that participates in a variety of cellular processes by deacetylating nonhistone substrates [59, 60]. Apart from its deacetylase activity, HDAC6 also includes a zinc-finger ubiquitin binding domain that modulates a wide range of physiological processes by interacting with proteins followed by induction of their degradations through the ubiquitin–proteasome system [59, 60]. As a result, HDAC6 plays pivotal roles in multiple pathological processes, including neurodegenerative diseases, cancers, and viral infections [59, 60]. Indeed, HDAC6 has been demonstrated to exert antiviral activities against numerous viruses, including swine enteric coronaviruses [61–63]. HDAC6 significantly restricts the replication of all four swine enteric coronaviruses SADS-CoV, PEDV, TGEV, and PDCoV [62, 63]. While the specific mechanism by which HDAC6 inhibits the replication of SADS-CoV, PEDV, and TGEV remains unidentified, the research on PDCoV reveals that HDAC6 interacts with PDCoV NSP8 and induces its degradation through the deacetylation at the lysine 46 (K46) and the ubiquitination at K58, thus restricting viral replication [62]. However, PDCoV NSP5 is able to cleave HDAC6 at glutamine 519 (Q519), which leads to a loss in its ability to degrade NSP8, to dampen its antiviral effect [63]. Interestingly, the NSP5 orthologs from SADS-CoV, PEDV, and TGEV also target HDAC6 at residue Q519 for cleavage, demonstrating that swine enteric coronaviruses share common strategy of NSP5-mediated cleavage to antagonize the antiviral activity of HDAC6 [63]. Future work will be needed to define whether HDAC6 also targets NSP8 orthologs from SADS-CoV, PEDV, and TGEV for proteasomal degradation to restrain viral replication (Figure 2).

## Innate immunity to SADS-CoV

### Type I and type III IFN inhibit SADS-CoV

Type I and type III IFN are the most critical effector molecules in the host antiviral innate immunity. Although SADS-CoV infection fails to induce IFNβ and IFNλ production robustly [64, 65], pretreatment of cells with IFNα, IFNδ8, and IFNλ3 can effectively inhibit SADS-CoV replication [66–68]. Interestingly, IFNα-mediated inhibition of SADS-CoV replication is dependent on the expression of the host factor tet methylcytosine dioxygenase 2 (TET2) [66]. TET2 protein is well-known for its catalytic activity in the conversion of methylcytosine to 5-hydroxymethylcytosine, and plays important roles in DNA repair, innate immunity, and inflammation [69, 70]. Knockout of TET2 compromises the antiviral effects of IFNα on SADS-CoV replication, which is correlated with the significant downregulation of key ISG including IFITM1 and IFITM3 [66]. TET2 was previously demonstrated to regulate IFITM3 promoter demethylation to promote IFITM3 expression [71]. However, it remains unknown whether TET2 facilitates type I IFN-mediated inhibition of SADS-CoV replication by regulating IFITM3 expression.

### Evasion of type I IFN-mediated antiviral immunity by SADS-CoV

**Figure 3:**
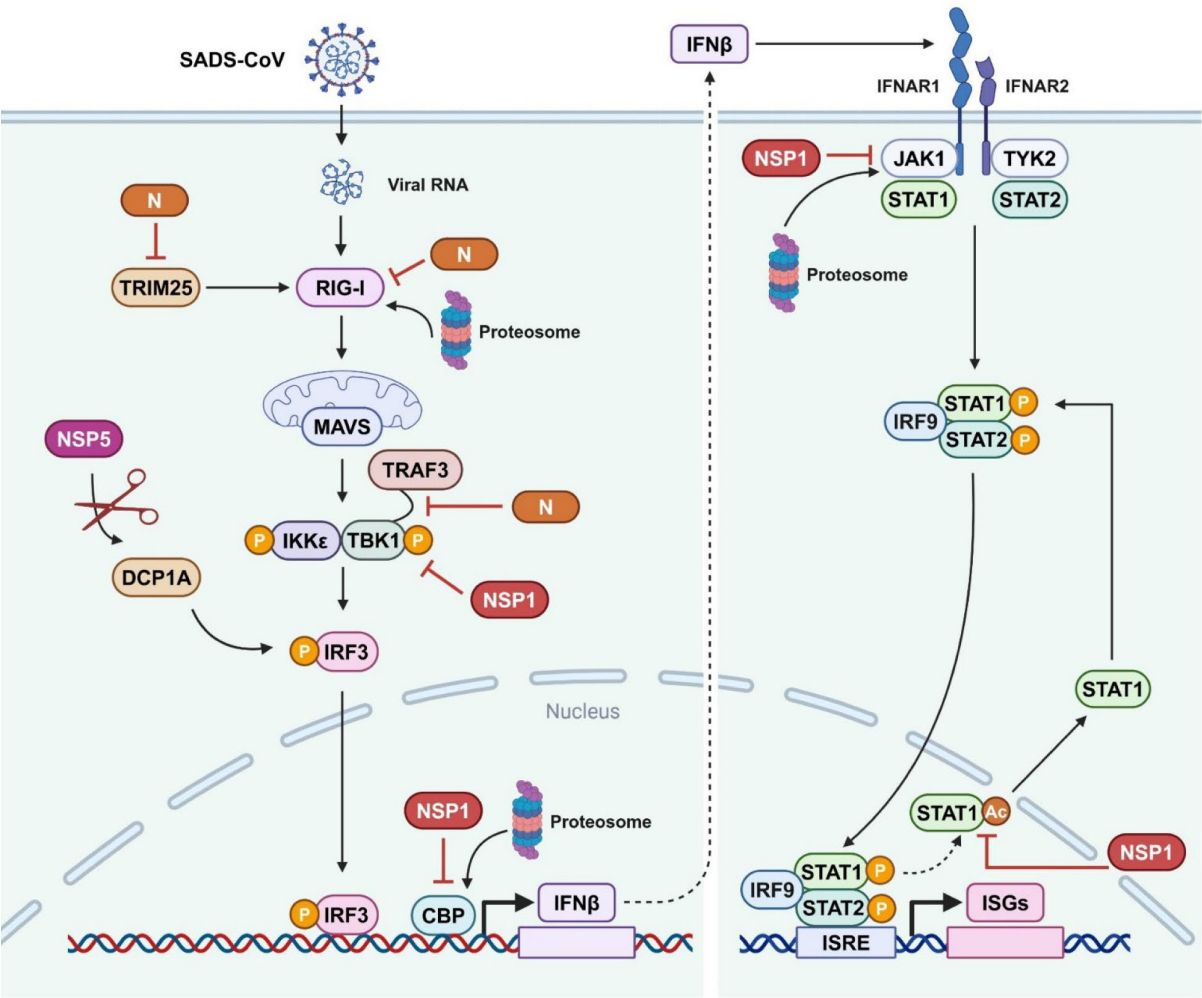
**Inhibition of type I IFN response by SADS-CoV**. SADS-CoV N protein inhibits IFNβ production by (1) inducing RIG-I degradation through ubiquitin proteasome pathway, (2) disrupting TRAF3-TBK1 interaction through interaction with TBK1 and IKKε, (3) interfering with TRIM25 oligomerization and TRIM25-RIG‑I interaction through interaction with TRIM25. SADS-CoV NSP1 inhibits IFNβ and ISG production by (1) inhibiting TBK1 phosphorylation, (2) inducing CBP degradation through the proteasome-dependent pathway, (3) triggering JAK1 degradation through the ubiquitin proteasome pathway, (4) suppressing STAT1 acetylation and dephosphorylation, blocking its nuclear export. SADS-CoV NSP5 inhibits IFNβ production by cleaving DCP1A through its protease activity. Figure created with BioRender.

Viruses have acquired numerous armaments to dampen host antiviral innate immunity during coevolution with their hosts. Thus, it is not surprising that SADS-CoV encodes multiple viral proteins, including N protein, NSP1, and NSP5, to inhibit type I IFN response in order to evade host antiviral innate immunity and favor its replication (Figure 3).

N proteins of swine enteric coronaviruses, including PEDV and PDCoV, have previously been demonstrated as the potent type I IFN antagonists that inhibit type I IFN production via distinct mechanisms [72, 73]. Consistent with this, SADS-CoV N protein was recently shown to suppress type I IFN production as well [74–76]. Mechanistically, SADS-CoV N protein employs multiple strategies to restrict type I IFN response [74–76]. First, SADS-CoV N protein interacts with RIG-I in an RNA-independent manner and induces its K27-, K48- and K63-linked ubiquitination, which leads to proteasome-dependent degradation of RIG-I and subsequent repression of Sendai virus (SeV)-triggered IFNβ production [74]. Second, SADS-CoV N protein interacts with TBK1 and IKKε, which disrupts the interaction between TRAF3 and TBK1, resulting in the reduction of SeV-mediated TBK1 activation and subsequent IFNβ production [75]. Lastly, SADS‑CoV N protein interacts with the coiled-coil dimerization (CCD) domain of tripartite motif-containing protein 25 (TRIM25), which inhibits TRIM25 oligomerization and interferes with the interaction of TRIM25 and RIG‑I, causing the suppression of TRIM25-mediated enhancement of RIG-I signalling and SeV-induced IFNβ production [76]. TRIM25 is an important host E3 ubiquitin ligase that regulates antiviral immunity by inducing RIG-I oligomerization through nondegradative K63-linked polyubiquitin to enhance RIG-I signalling [77]. Interestingly, TRIM25 can enhance the antiviral activity of zinc-finger antiviral protein (ZAP) by mediating both K48- and K63-linked polyubiquitination of ZAP [78, 79]. Furthermore, TRIM25 has been demonstrated to interact with influenza virus ribonucleoproteins to inhibit the initiation of RNA chain elongation, thus restricting viral replication [80]. However, the specific mechanism of TRIM25-mediated inhibition of SADS-CoV replication remains unknown.

The NSP1 proteins from swine enteric alphacoronaviruses, including TGEV, PEDV, and SADS-CoV, have a shared function in the inhibition of type I IFN signaling [81]. SADS-CoV NSP1 inhibits TBK1 phosphorylation by disrupting the interaction between TBK1 and the Ub protein, and specifically induces the degradation of CREB‐binding protein (CBP) through the proteasome-dependent pathway, thus preventing the formation of IFN transcriptional enhancer and suppressing IFNβ production [82]. Intriguingly, SADS-CoV NSP1 induces K11- and K48-linked JAK1 polyubiquitination and triggers JAK1 degradation through the ubiquitin proteasome pathway [83]. Moreover, SADS-CoV NSP1 inhibits STAT1 acetylation and dephosphorylation by inducing CBP degradation, which blocks STAT1 export from the nucleus to the cytoplasm and restricts ISG expression [83]. These findings reveal two novel mechanisms by which SADS-CoV NSP1 restrains both the RLR signalling pathway and the JAK-STAT signalling pathway to evade type I IFN-mediated antiviral innate immunity.

Coronavirus NSP5, also called 3C-like protease, is not only capable of cleaving viral polypeptides to facilitate viral replication, but also cutting immune-related molecules to evade host antiviral immunity. Specifically, SADS-CoV NSP5 has been demonstrated to target and cleave mRNA-decapping enzyme 1a (DCP1A) to antagonize the type I IFN signaling pathway [84]. DCP1A is well-recognized for its central role in removing the 5′-methylguanosine cap from eukaryotic mRNA, and has recently been identified as an antiviral ISG against several viruses [85–87]. SADS-CoV NSP5 cleaves DCP1A via its protease activity, which is dependent on the critical amino acid residues of histidine at 41 and cystine at 144, to inhibit IRF3 and NF-κB signalling pathways, thus decreasing the expression of IFNβ and proinflammatory cytokines [84]. Interestingly, A DCP1A variant with a mutation in glutamine at 343 is resistant to NSP5-mediated cleavage, which displays a stronger inhibitory effect on SADS-CoV replication than wild-type protein [84]. Notably, the NSP5 proteins from different coronaviruses, including SADS-CoV, PDCoV, SARS-CoV, SARS-CoV-2, and MERS-CoV, exhibit similar cleavage activities on DCP1A in infected cells, implicating that NSP5-mediated DCP1A cleavage might be a conserved mechanism by which coronaviruses avoid host antiviral innate immune responses [84, 86].

### Evasion of type III IFN-mediated antiviral Immunity by SADS-CoV

**Figure 4:**
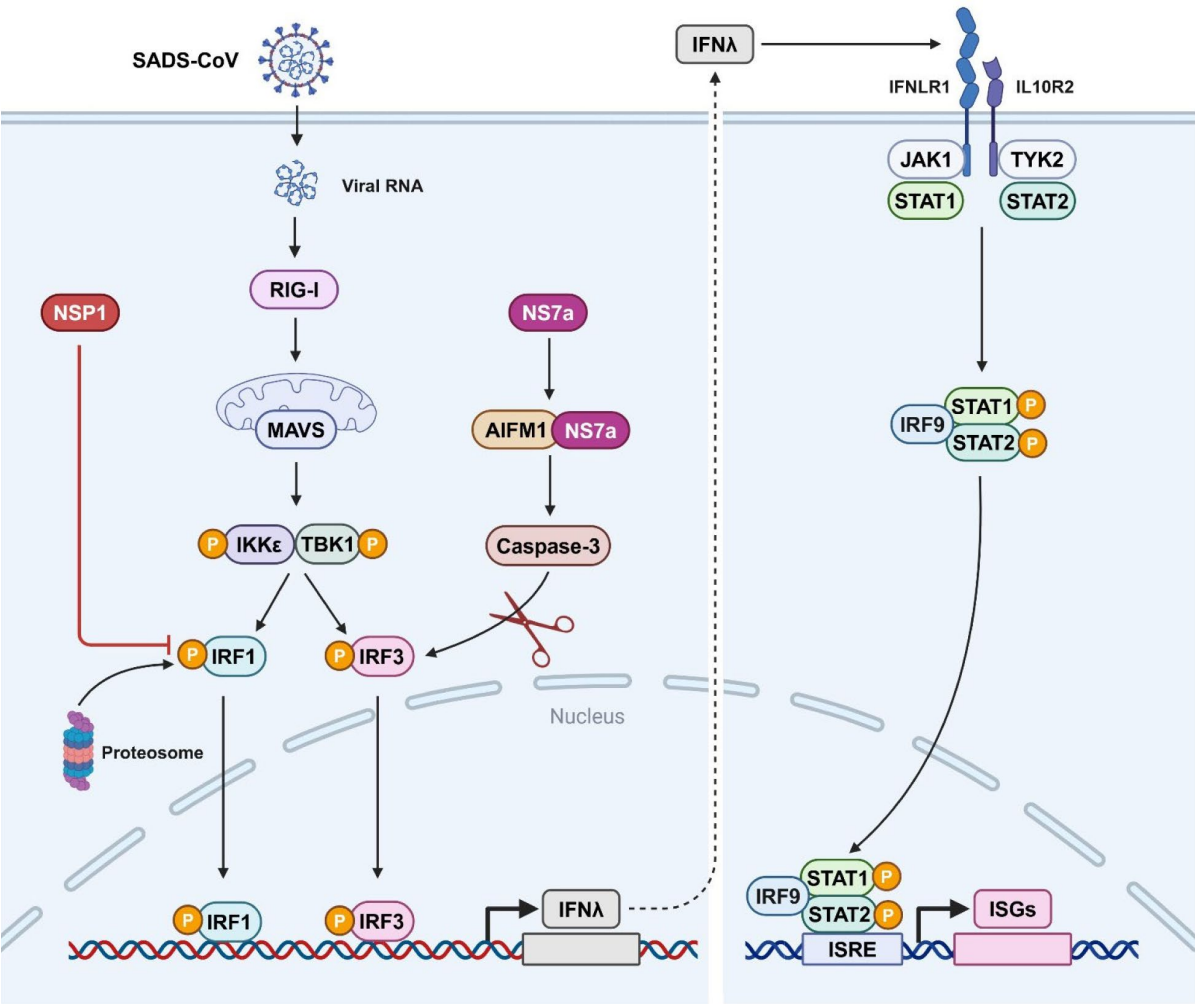
**Inhibition of type III IFN response by SADS-CoV**. SADS-CoV NSP1 inhibits IFNλ production by inducing IRF1 degradation through the ubiquitin–proteasome pathway. SADS-CoV NS7a inhibits IFNλ production by cleaving IRF3 through activation of caspase-3 by interacting with AIFM1. Figure created with BioRender.

In contrast to the ubiquitous expression of type I IFN receptors, type III IFN receptors are restricted to the mucosal epithelium [88]. Consistent with this, intestinal epithelial cells have recently been shown to express extremely low levels of type I IFN receptors, but produce high levels of type III IFN, thereby triggering robust type III IFN-mediated antiviral immune response against enteric viruses [89]. Therefore, type III IFN-mediated antiviral immunity serves as the primary defence strategy against viruses that replicate in intestinal epithelial cells. Nevertheless, enteric viruses have evolved to employ multiple strategies to evade type III IFN-mediated antiviral immune response. In the case of SADS-CoV, it employs two viral proteins, NSP1 and NS7a, to inhibit the type III IFN response to promote viral replication [65, 67]. SADS-CoV NSP1 prevents poly(I:C)-induced nuclear translocation of IRF1 and induces its degradation through the ubiquitin–proteasome pathway, thus reducing IFNλ expression (Figure 4). However, SADS-CoV NSP1 does not directly interact with IRF1, implicating that it might recruit specific host E3 ubiquitin ligase to degrade IRF1. Additionally, SADS-CoV NS7a interacts with apoptosis-inducing factor mitochondria associated 1 (AIFM1) to activate caspase-3, which cleaves IRF3, thereby inhibiting IFNλ production (Figure 4).

## SADS-CoV manipulation of autophagy for replication

Autophagy is an evolutionarily conserved catabolic cellular process by which the cellular components including aggregated proteins and damaged organelles are transported to the lysosome for degradation [90, 91]. It is a highly orchestrated cellular process that is tightly regulated by more than 30 autophagy-related genes (ATG) and encompasses four sequential steps: autophagy initiation, elongation and closure of the autophagic membrane, fusion of autophagosome with lysosome, and autophagosome degradation [90, 91]. Under various physiological and pathological conditions including cell development and differentiation, starvation, hypoxia, and virus infection, the highly conserved protein kinase mammalian target of rapamycin (mTOR) is inhibited, and autophagy is thus induced to maintain cellular homeostasis [90, 91]. Inhibition of mTOR signalling allows the formation of the Unc-51 like autophagy activating kinase 1 (ULK1)-ATG13-FAK family kinase-interacting protein of 200 kDa (FIP200)-ATG101 complex, which phosphorylates and activates the downstream Beclin1-ATG14L-Vacuolar protein sorting 15 (VPS15)-VPS34 complex, leading to the formation of phagophore, an isolated double-membraned vesicle that encapsulates cytosolic components inside itself [92, 93]. The elongation and closure step depends on the ATG16L1-ATG5-ATG12 complex, which facilitates microtubule-associated proteins 1 light chain 3-I (LC3-I) lipidation on the phagophore membrane to form LC3-II, resulting in the formation of autophagosome [92, 93]. Subsequently, the pleckstrin homology domain-containing protein family member 1 (PLEKHM1) tethers autophagosomes by binding to LC3 with lysosomes by interacting with RAB7, and then the fusion between autophagosome and lysosome is triggered by the tail-anchored SNAP receptor (SNARE) syntaxin 17 (STX17), which forms a structure termed autolysosome [92]. Finally, the contents within the autolysosomes are subjected to acidification, and then degraded by lysosomal hydrolases and recycled back to the cytosol [94].

Numerous studies have demonstrated autophagy as a critical branch of the host antiviral innate immunity, which is capable of degrading virions, viral proteins, or even host factors essential for viral replication, and cooperates with host PRR signalling to promote IFN production to inhibit viral replication [92, 95–99]. However, viruses have evolved to employ numerous strategies to evade autophagy or even harness autophagy for their benefits [100–105]. Similar to other swine enteric coronaviruses [106–108], the newly emerged SADS-CoV is also a master that is very good at utilizing autophagy pathway to facilitate viral replication [109, 110]. SADS-CoV infection triggers autophagy not only in Vero E6 cells, swine testis (ST) cells, IPI-2I, and porcine ileum epithelial cells IPI-FX in vitro, but also in the ileal tissues of piglets in vivo [109, 110]. Pharmacological induction of autophagy significantly promotes SADS-CoV replication, while pharmacological inhibition of autophagy or knockdown of autophagy-related proteins compromises SADS-CoV replication, demonstrating a proviral role for autophagy during SADS-CoV infection [109, 110]. Mechanistically, SADS-CoV could utilize two distinct means to induce autophagy to promote viral replication: (1) SADS-CoV infection results in a reduction in the expression of the negative regulator of autophagy, integrin a3 (ITGA3), which inhibits AKT and mTOR phosphorylation, thus inducing autophagy; (2) SADS-CoV infection produces viral membrane-associated PLP2 (PLP2-TM) that interacts with glucose-regulated protein of 78 kDa (GRP78) to form a complex, which activates ER stress response and the inositol-requiring enzyme 1 (IRE1) signalling pathway, then the JNK-Beclin 1 adaptors bridge the ER stress response and autophagy, demonstrating the critical role for IRE1-JNK-Beclin 1 signalling pathway in SADS-CoV-induced autophagy. However, the specific molecular mechanisms by which (1) SADS-CoV represses ITGA3 expression, (2) ITGA3 inhibits AKT phosphorylation, and (3) SADS-CoV interplays with the autophagy pathway warrant further investigations.

## SADS-CoV manipulation of apoptosis for replication

Cell death is a normal but critical physiological process in all living organisms by which senescent and damaged cells are removed to maintain cell homeostasis. Of the three most well understood cell death pathways (apoptosis, pyroptosis, and necroptosis), apoptosis was the first to be identified [111, 112]. Apoptosis is a conserved programmed cell death across the animal kingdom, which is induced by various physiological and pathological stimuli and characterized by decreased cell size, membrane blebbing, chromatin condensation, nuclear fragmentation, and the formation of apoptotic bodies [113, 114]. Apoptosis is initiated by two main pathways known as the intrinsic and extrinsic pathways. The intrinsic pathway is triggered by intracellular stressors including growth factors, nutrient deprivation, DNA damage, and ER stress, and is characterized by mitochondrial outer membrane permeabilization (MOMP) and the release of cytochrome c into the cytosol, which activates the cascade of caspase-9 signalling to induce cell death [113, 114]. In contrast, the extrinsic pathway is initiated by recognition of extracellular signals by death receptors including Fas receptor (FasR), TNF receptor 1 and 2 (TNFR1/2), and TRAIL receptors DR4 and DR5, which activates the cascade of caspase-8 signalling to induce cell death [113, 114].

Accumulative evidence demonstrates that the PRR of the mammalian innate immune system can activate cell apoptotic pathway [115, 116], suggesting that apoptosis is an essential branch of host innate defence mechanisms against viral infections [117, 118]. However, numerous viruses have evolved to adopt diverse mechanisms to hijack the apoptosis pathway to facilitate viral replication [119–122]. Therefore, it is not surprising that, similar to other swine enteric coronaviruses [106–108, 119], the newly emerged SADS-CoV is also capable of inducing apoptosis to favour viral fitness [67, 123]. SADS-CoV infection induces apoptosis not only in Vero E6, IPI-2I, IPI-FX, and HeLa cells in vitro, but also in the ileal and jejunum tissues of piglets in vivo [67, 123]. SADS-CoV infection activates the apoptosis initiator caspase-8, which in turn cleaves the proapoptotic BH3-interacting domain death agonist (Bid), cleaved Bid then activates caspase-9, leading to the induction of apoptosis via both the intrinsic and extrinsic pathways [123]. Importantly, both caspase-8 and caspase-9 inhibitors severely block SADS-CoV-induced apoptosis, and thus repress viral replication [123]. Interestingly, activation of the extracellular signal-regulated kinase 1/2 (ERK1/2) signalling pathway is required for SADS-CoV-induced apoptosis, and blocking this pathway significantly inhibits viral replication [124]. Moreover, SADS-CoV NS7a has been demonstrated to activate AIFM1 and caspase-3, which are transported into the nucleus to induce apoptosis, thereby promoting viral replication [67]. Remarkably, the caspase-3 inhibitor Z-DEVD-FMK significantly reduces SADS-CoV replication in the intestinal tissues and elevates the survival rate of infected piglets, demonstrating apoptosis inhibitors as the promising therapeutic drugs for the prevention and control of SADS-CoV infection [67].

## Conclusions and future perspectives

SADS-CoV is a newly emerged swine enteropathogenic coronavirus that infects a wide range of cells from human and diverse animals, holding potential cross‐species transmission risks. The intracellular antiviral immunity is the first line of the host defence system that combats viral infection in an IFN-independent and -dependent fashion. Three host restriction factors, CH25H, TMEM53, and HDAC6, have been demonstrated to target different stages of viral life cycle to restrict SADS-CoV replication (Figure 2). Moreover, both type I and type III IFN are able to potently restrain SADS-CoV replication. However, SADS-CoV has evolved to evade type I- and type III-mediated innate immune responses by encoding multiple viral proteins, including N protein, NSP1, NSP5, and NS7a (Figures 3 and 4). In addition, SADS-CoV has the ability to hijack the autophagy and apoptosis pathways to favour its fitness. Altogether, the interplay between SADS-CoV infection and the host intrinsic and innate immunity is a complex and competitive balance process.

Although much progress has been made to reveal the complicated relationship between SADS-CoV and the host intracellular antiviral immunity in the last few years, it is still not well characterized and needs extensive investigations in the future. As a major topic in future studies, the information on cell-intrinsic immunity is still very limited, which warrants in-depth investigations. To date, there are only three host restriction factors identified to restrain SADS-CoV replication, much more remain to be discovered. While the mutual relationship between SADS-CoV infection and type I and type III IFN-mediated innate immunity has recently been revealed, the interplay of SADS-CoV and type II IFN-mediated innate immunity remains to be clarified. Furthermore, little is known about the roles of the immune molecules downstream of the IFN signalling pathways, such as ISG, during SADS-CoV replication. Additionally, whether other innate immune responses including DNA damage response, ER stress, stress granules, complement activation, and RNA interference are involved in immune control of SADS-CoV infection is unknown and is thus worthy of future attention. Finally, in-depth and intensive studies on host intrinsic and innate immune factors will undoubtedly promote the identification of new targets for the development of intervention strategies against SADS-CoV infection.

## Data Availability

Not applicable.

## References

[1] V’kovski P, Kratzel A, Steiner S, Stalder H, Thiel V (2021) Coronavirus biology and replication: implications for SARS-CoV-2. Nat Rev Microbiol 19:155–170. 10.1038/s41579-020-00468-6 33116300 10.1038/s41579-020-00468-6 PMC7592455

[2] Kong F, Jia H, Xiao Q, Fang L, Wang Q (2024) Prevention and control of swine enteric coronaviruses in China: a review of vaccine development and application. Vaccines 12:11. 10.3390/vaccines12010011 10.3390/vaccines12010011 PMC10820180 38276670

[3] Turlewicz-Podbielska H, Pomorska-Mól M (2021) Porcine coronaviruses: Overview of the state of the art. Virol Sin 36:833–851. 10.1007/s12250-021-00364-0 33723809 10.1007/s12250-021-00364-0 PMC7959302

[4] Pan Y, Tian X, Qin P, Wang B, Zhao P, Yang Y-L, Wang L, Wang D, Song Y, Zhang X, Huang Y-W (2017) Discovery of a novel swine enteric alphacoronavirus (SeACoV) in southern China. Vet Microbiol 211:15–21. 10.1016/j.vetmic.2017.09.020 29102111 10.1016/j.vetmic.2017.09.020 PMC7117260

[5] Gong L, Li J, Zhou Q, Xu Z, Chen L, Zhang Y, Xue C, Wen Z, Cao Y (2017) A new bat-HKU2–like coronavirus in swine, China, 2017. Emerg Infect Dis 23:1607–1609. 10.3201/eid2309.170915 28654418 10.3201/eid2309.170915 PMC5572857

[6] Zhou P, Fan H, Lan T, Lou YX, Shi WF, Zhang W, Zhu Y, Zhang YW, Xie QM, Mani S, Zheng XS, Li B, Li JM, Guo H, Pei GQ, An XP, Chen JW, Zhou L, Mai KJ, et al. (2018) Fatal swine acute diarrhoea syndrome caused by an HKU2-related coronavirus of bat origin. Nature 556:255–259. 10.1038/s41586-018-0010-9 29618817 10.1038/s41586-018-0010-9 PMC7094983

[7] Xu Z, Zhang Y, Gong L, Huang L, Lin Y, Qin J, Du Y, Zhou Q, Xue C, Cao Y (2019) Isolation and characterization of a highly pathogenic strain of Porcine enteric alphacoronavirus causing watery diarrhoea and high mortality in newborn piglets. Transbound Emerg Dis 66:119–130. 10.1111/tbed.12992 30103259 10.1111/tbed.12992 PMC7168553

[8] Zhou L, Li QN, Su JN, Chen GH, Wu ZX, Luo Y, Wu RT, Sun Y, Lan T, Ma JY (2019) The re-emerging of SADS-CoV infection in pig herds in Southern China. Transbound Emerg Dis 66:2180–2183. 10.1111/tbed.13270 31207129 10.1111/tbed.13270 PMC7168562

[9] Li K, Li H, Bi Z, Gu J, Gong W, Luo S, Zhang F, Song D, Ye Y, Tang Y (2018) Complete genome sequence of a novel swine acute diarrhea syndrome coronavirus, CH/FJWT/2018, isolated in Fujian, China, in 2018. Microbiol Resour Announc 7:e01259-18. 10.1128/mra.01259-18 30533848 10.1128/MRA.01259-18 PMC6284080

[10] Sun Y, Xing J, Xu Z, Gao H, Xu S, Liu J, Zhu D, Guo Y, Yang B, Chen X, Zheng Z, Wang H, Lang G, Holmes EC, Zhang G (2022) Re-emergence of severe acute diarrhea syndrome coronavirus (SADS-CoV) in Guangxi, China, 2021. J Infect 85:e130–e133. 10.1016/j.jinf.2022.08.020 36002051 10.1016/j.jinf.2022.08.020 PMC9393104

[11] Zhang T, Yao J, Yang Z, Wang J, Yang K, Yao L (2024) Re-emergence of severe acute diarrhea syndrome coronavirus (SADS-CoV) in Henan, central China, 2023. Vet Microbiol 292:110049. 10.1016/j.vetmic.2024.110049 38493699 10.1016/j.vetmic.2024.110049

[12] Liu C, Huang W, He X, Feng Z, Chen Q (2024) Research advances on swine acute diarrhea syndrome coronavirus. Animals 14:448. 10.3390/ani14030448 38338091 10.3390/ani14030448 PMC10854734

[13] Le YY, Yu JQ, Huang YW (2020) Swine enteric alphacoronavirus (swine acute diarrhea syndrome coronavirus): an update three years after its discovery. Virus Res 285:198024. 10.1016/j.virusres.2020.198024 32482591 10.1016/j.virusres.2020.198024 PMC7229464

[14] Kim J, Yoon J, Park JE (2022) Furin cleavage is required for swine acute diarrhea syndrome coronavirus spike protein-mediated cell–cell fusion. Emerg Microbes Infect 11:2176–2183. 10.1080/22221751.2022.2114850 35976165 10.1080/22221751.2022.2114850 PMC9518401

[15] Zhu Z, Han Y, Gong M, Sun B, Zhang R, Ding Q (2024) Establishment of replication-competent vesicular stomatitis virus recapitulating SADS-CoV entry. J Virol 98:e01957-23. 10.1128/jvi.01957-23 38557247 10.1128/jvi.01957-23 PMC11092325

[16] Yang Y-L, Wang B, Li W, Cai H-L, Qian Q-Y, Qin Y, Shi F-S, Bosch B-J, Huang Y-W (2024) Functional dissection of the spike glycoprotein S1 subunit and identification of cellular cofactors for regulation of swine acute diarrhea syndrome coronavirus entry. J Virol 98:e00139-24. 10.1128/jvi.00139-24 38501663 10.1128/jvi.00139-24 PMC11019839

[17] Yang Y-L, Qin P, Wang B, Liu Y, Xu G-H, Peng L, Zhou J, Zhu SJ, Huang Y-W (2019) Broad cross-species infection of cultured cells by bat HKU2-related swine acute diarrhea syndrome coronavirus and identification of its replication in murine dendritic cells in vivo highlight its potential for diverse interspecies transmission. J Virol 93:e01448-19. 10.1128/jvi.01448-19 31554686 10.1128/JVI.01448-19 PMC6880172

[18] Edwards CE, Yount BL, Graham RL, Leist SR, Hou YJ, Dinnon KH, Sims AC, Swanstrom J, Gully K, Scobey TD, Cooley MR, Currie CG, Randell SH, Baric RS (2020) Swine acute diarrhea syndrome coronavirus replication in primary human cells reveals potential susceptibility to infection. Proc Natl Acad Sci USA 117:26915–26925. 10.1073/pnas.2001046117 33046644 10.1073/pnas.2001046117 PMC7604506

[19] Luo Y, Chen Y, Geng R, Li B, Chen J, Zhao K, Zheng XS, Zhang W, Zhou P, Lou YX, Shi ZL (2021) Broad cell tropism of SADS-CoV in vitro implies its potential cross-species infection risk. Virol Sin 36:559–563. 10.1007/s12250-020-00321-3 33201410 10.1007/s12250-020-00321-3 PMC7670973

[20] Mei XQ, Qin P, Le YY, Liao M, Liang QZ, Zhao Z, Shi FS, Wang B, Huang YW (2022) First evidence that an emerging mammalian alphacoronavirus is able to infect an avian species. Transbound Emerg Dis 69:e2006–e2019. 10.1111/tbed.14535 35340130 10.1111/tbed.14535

[21] Guo Z, Jin Q, Li P, Xing G, Lu Q, Zhang G (2023) Potential cross-species transmission risks of emerging swine enteric coronavirus to human beings. J Med Virol 95:e28919. 10.1002/jmv.28919 37386904 10.1002/jmv.28919

[22] Chen Y, Liu X, Zheng J, Yang L, Luo Y, Yao Y, Liu M, Xie T, Lin H (2023) N-linked glycoproteins and host proteases are involved in swine acute diarrhea syndrome coronavirus entry. J Virol 97:e00916-23. 10.1128/jvi.00916-23 37772826 10.1128/jvi.00916-23 PMC10617469

[23] Han Y, Ma Y, Wang Z, Feng F, Feng H, Ma J, Ye R, Zhang R (2024) TMPRSS13 promotes the cell entry of swine acute diarrhea syndrome coronavirus. J Med Virol 96:e29712. 10.1002/jmv.29712 38808555 10.1002/jmv.29712

[24] Yang QY, Le YY, Tang YX, Qin P, Wang G, Xie JY, Chen SX, Ding C, Huang YW, Zhu SJ (2022) Bile acids promote the caveolae-associated entry of swine acute diarrhea syndrome coronavirus in porcine intestinal enteroids. PLoS Pathog 18:e1010620. 10.1371/journal.ppat.1010620 35696443 10.1371/journal.ppat.1010620 PMC9249351

[25] Pradeu T, Thomma BPHJ, Girardin SE, Lemaitre B (2024) The conceptual foundations of innate immunity: taking stock 30 years later. Immunity 57:613–631. 10.1016/j.immuni.2024.03.007 38599162 10.1016/j.immuni.2024.03.007

[26] Majdoul S, Compton AA (2022) Lessons in self-defence: inhibition of virus entry by intrinsic immunity. Nat Rev Immunol 22:339–352. 10.1038/s41577-021-00626-8 34646033 10.1038/s41577-021-00626-8 PMC8511856

[27] Alandijany T (2019) Host intrinsic and innate intracellular immunity during herpes simplex virus type 1 (HSV-1) infection. Front Microbiol 10:2611. 10.3389/fmicb.2019.02611 31781083 10.3389/fmicb.2019.02611 PMC6856869

[28] Randow F, MacMicking JD, James LC (2013) Cellular self-defense: how cell-autonomous immunity protects against pathogens. Science 340:701–706. 10.1126/science.1233028 23661752 10.1126/science.1233028 PMC3863583

[29] Yan N, Chen ZJ (2012) Intrinsic antiviral immunity. Nat Immunol 13:214–222. 10.1038/ni.2229 22344284 10.1038/ni.2229 PMC3549670

[30] Bottermann M, James LC (2018) Intracellular antiviral immunity. Adv Virus Res 100:309–354. 10.1016/bs.aivir.2018.01.002 29551141 10.1016/bs.aivir.2018.01.002 PMC7172442

[31] Savidis G, Perreira JM, Portmann JM, Meraner P, Guo Z, Green S, Brass AL (2016) The IFITMs inhibit Zika virus replication. Cell Rep 15:2323–2330. 10.1016/j.celrep.2016.05.074 27268505 10.1016/j.celrep.2016.05.074

[32] Yu J, Li M, Wilkins J, Ding S, Swartz TH, Esposito AM, Zheng Y-M, Freed EO, Liang C, Chen BK, Liu S-L (2015) IFITM proteins restrict HIV-1 infection by antagonizing the envelope glycoprotein. Cell Rep 13:145–156. 10.1016/j.celrep.2015.08.055 26387945 10.1016/j.celrep.2015.08.055 PMC4602366

[33] Wang J, Wang CF, Ming SL, Li GL, Zeng L, Di WM, Su BQ, Wang Q, Yang GY, Chu BB (2020) Porcine IFITM1 is a host restriction factor that inhibits pseudorabies virus infection. Int J Biol Macromol 151:1181–1193. 10.1016/j.ijbiomac.2019.10.162 31743714 10.1016/j.ijbiomac.2019.10.162 PMC7102536

[34] Sun X, Zeng H, Kumar A, Belser JA, Maines TR, Tumpey TM (2016) Constitutively expressed IFITM3 protein in human endothelial cells poses an early infection block to human influenza viruses. J Virol 90:11157–11167. 10.1128/JVI.01254-16 27707929 10.1128/JVI.01254-16 PMC5126373

[35] Xie J, Bi Y, Xu S, Han Y, Idris A, Zhang H, Li X, Bai J, Zhang Y, Feng R (2020) Host antiviral protein IFITM2 restricts pseudorabies virus replication. Virus Res 287:198105. 10.1016/j.virusres.2020.198105 32745511 10.1016/j.virusres.2020.198105 PMC7834200

[36] Wang J, Zeng L, Zhang L, Guo ZZ, Lu SF, Ming SL, Li GL, Wan B, Tian KG, Yang GY, Chu BB (2017) Cholesterol 25-hydroxylase acts as a host restriction factor on pseudorabies virus replication. J Gen Virol 98:1467–1476. 10.1099/jgv.0.000797 28631596 10.1099/jgv.0.000797

[37] Doms A, Sanabria T, Hansen JN, Altan-Bonnet N, Holm GH (2018) 25-Hydroxycholesterol production by the cholesterol-25-hydroxylase interferon-stimulated gene restricts mammalian reovirus infection. J Virol 92:e01047-18. 10.1128/JVI.01047-18 29950420 10.1128/JVI.01047-18 PMC6146694

[38] Liu D, Shi D, Shi H, Zhang L, Zhang J, Zeng M, Feng T, Yang X, Zhang X, Chen J, Jing Z, Ji Z, Zhang J, Feng L (2023) Cholesterol 25-hydroxylase suppresses swine acute diarrhea syndrome coronavirus infection by blocking spike protein-mediated membrane fusion. Viruses 15:2406. 10.3390/v15122406 38140647 10.3390/v15122406 PMC10747074

[39] Lo Cigno I, De Andrea M, Borgogna C, Albertini S, Landini MM, Peretti A, Johnson KE, Chandran B, Landolfo S, Gariglio M (2015) The nuclear DNA sensor IFI16 acts as a restriction factor for human papillomavirus replication through epigenetic modifications of the viral promoters. J Virol 89:7506–7520. 10.1128/jvi.00013-15 25972554 10.1128/JVI.00013-15 PMC4505635

[40] Hotter D, Bosso M, Jønsson KL, Krapp C, Stürzel CM, Das A, Littwitz-Salomon E, Berkhout B, Russ A, Wittmann S, Gramberg T, Zheng Y, Martins LJ, Planelles V, Jakobsen MR, Hahn BH, Dittmer U, Sauter D, Kirchhoff F (2019) IFI16 targets the transcription factor Sp1 to suppress HIV-1 transcription and latency reactivation. Cell Host Microbe 25:858-872.e13. 10.1016/j.chom.2019.05.002 31175045 10.1016/j.chom.2019.05.002 PMC6681451

[41] Howard TR, Lum KK, Kennedy MA, Cristea IM (2022) The nuclear DNA sensor IFI16 indiscriminately binds to and diminishes accessibility of the HSV-1 genome to suppress infection. mSystems 7:e00198-22. 10.1128/msystems.00198-22 35575489 10.1128/msystems.00198-22 PMC9239196

[42] Ames J, Yadavalli T, Suryawanshi R, Hopkins J, Agelidis A, Patil C, Fredericks B, Tseng H, Valyi-Nagy T, Shukla D (2021) OPTN is a host intrinsic restriction factor against neuroinvasive HSV-1 infection. Nat Commun 12:5401. 10.1038/s41467-021-25642-z 34518549 10.1038/s41467-021-25642-z PMC8437952

[43] Yao YL, Luo Y, Wang Q, Geng R, Chen Y, Liu MQ, Li B, Chen J, Wu CG, Jia JK, Luo JY, He YT, Jiang TT, Zhu Y, Hu B, Zhou P, Shi ZL (2023) Identification of TMEM53 as a novel SADS-CoV restriction factor that targets viral RNA-dependent RNA polymerase. Emerg Microbes Infect 12:2249120. 10.1080/22221751.2023.2249120 37584551 10.1080/22221751.2023.2249120 PMC10467534

[44] Zhu H, Zheng C (2020) The race between host antiviral innate immunity and the immune evasion strategies of herpes simplex virus 1. Microbiol Mol Biol Rev 84:e00099-20. 10.1128/mmbr.00099-20 32998978 10.1128/MMBR.00099-20 PMC7528619

[45] Rehwinkel J, Gack MU (2020) RIG-I-like receptors: their regulation and roles in RNA sensing. Nat Rev Immunol 20:537–551. 10.1038/s41577-020-0288-3 32203325 10.1038/s41577-020-0288-3 PMC7094958

[46] Yoneyama M, Kato H, Fujita T (2024) Physiological functions of RIG-I-like receptors. Immunity 57:731–751. 10.1016/j.immuni.2024.03.003 38599168 10.1016/j.immuni.2024.03.003

[47] Schoggins JW (2019) Interferon-stimulated genes: what do they all do? Annu Rev Virol 6:567–584. 10.1146/annurev-virology-092818-015756 31283436 10.1146/annurev-virology-092818-015756

[48] Park A, Iwasaki A (2020) Type I and type III interferons—induction, signaling, evasion, and application to combat COVID-19. Cell Host Microbe 27:870–878. 10.1016/j.chom.2020.05.008 32464097 10.1016/j.chom.2020.05.008 PMC7255347

[49] Lazear HM, Schoggins JW, Diamond MS (2019) Shared and distinct functions of type I and type III interferons. Immunity 50:907–923. 10.1016/j.immuni.2019.03.025 30995506 10.1016/j.immuni.2019.03.025 PMC6839410

[50] Zhao J, Chen J, Li M, Chen M, Sun C (2020) Multifaceted functions of CH25H and 25HC to modulate the lipid metabolism, immune responses, and broadly antiviral activities. Viruses 12:727. 10.3390/v12070727 32640529 10.3390/v12070727 PMC7411728

[51] Schoggins JW, Randall G (2013) Lipids in innate antiviral defense. Cell Host Microbe 14:379–385. 10.1016/j.chom.2013.09.010 24139397 10.1016/j.chom.2013.09.010 PMC3850052

[52] Cao Q, Liu Z, Xiong Y, Zhong Z, Ye Q (2020) Multiple roles of 25-hydroxycholesterol in lipid metabolism, antivirus process, inflammatory response, and cell survival. Oxid Med Cell Longev 2020:8893305. 10.1155/2020/8893305 33274010 10.1155/2020/8893305 PMC7695496

[53] Zhang Y, Song Z, Wang M, Lan M, Zhang K, Jiang P, Li Y, Bai J, Wang XW (2019) Cholesterol 25-hydroxylase negatively regulates porcine intestinal coronavirus replication by the production of 25-hydroxycholesterol. Vet Microbiol 231:129–138. 10.1016/j.vetmic.2019.03.004 30955800 10.1016/j.vetmic.2019.03.004 PMC7117535

[54] Ke W, Wu X, Fang P, Zhou Y, Fang L, Xiao S (2021) Cholesterol 25-hydroxylase suppresses porcine deltacoronavirus infection by inhibiting viral entry. Virus Res 295:198306. 10.1016/j.virusres.2021.198306 33476696 10.1016/j.virusres.2021.198306 PMC7833861

[55] Schirmer EC, Florens L, Guan T, Yates JRI, Gerace L (2003) Nuclear membrane proteins with potential disease links found by subtractive proteomics. Science 301:1380–1382 12958361 10.1126/science.1088176

[56] Malik P, Korfali N, Srsen V, Lazou V, Batrakou DG, Zuleger N, Kavanagh DM, Wilkie GS, Goldberg MW, Schirmer EC (2010) Cell-specific and lamin-dependent targeting of novel transmembrane proteins in the nuclear envelope. Cell Mol Life Sci 67:1353–1369. 10.1007/s00018-010-0257-2 20091084 10.1007/s00018-010-0257-2 PMC2839517

[57] Korfali N, Srsen V, Waterfall M, Batrakou DG, Pekovic V, Hutchison CJ, Schirmer EC (2011) A flow cytometry-based screen of nuclear envelope transmembrane proteins identifies NET4/tmem53 as involved in stress-dependent cell cycle withdrawal. PLoS One 6:e18762. 10.1371/journal.pone.0018762 21533191 10.1371/journal.pone.0018762 PMC3077400

[58] Guo L, Iida A, Bhavani GSL, Gowrishankar K, Wang Z, Xue JY, Wang J, Miyake N, Matsumoto N, Hasegawa T, Iizuka Y, Matsuda M, Nakashima T, Takechi M, Iseki S, Yambe S, Nishimura G, Koseki H, Shukunami C, Girisha KM, Ikegawa S (2021) Deficiency of TMEM53 causes a previously unknown sclerosing bone disorder by dysregulation of BMP-SMAD signaling. Nat Commun 12:2046. 10.1038/s41467-021-22340-8 33824347 10.1038/s41467-021-22340-8 PMC8024261

[59] Zheng K, Jiang Y, He Z, Kitazato K, Wang Y (2017) Cellular defence or viral assist: the dilemma of HDAC6. J Gen Virol 98:322–337. 10.1099/jgv.0.000679 27959772 10.1099/jgv.0.000679

[60] Zhu Y, Feng M, Wang B, Zheng Y, Jiang D, Zhao L, Mamun MAA, Kang H, Nie H, Zhang X, Guo N, Qin S, Wang N, Liu H, Gao Y (2023) New insights into the non-enzymatic function of HDAC6. Biomed Pharmacother 161:114438. 10.1016/j.biopha.2023.114438 37002569 10.1016/j.biopha.2023.114438

[61] Qu M, Zhang H, Cheng P, Wubshet AK, Yin X, Wang X, Sun Y (2023) Histone deacetylase 6’s function in viral infection, innate immunity, and disease: latest advances. Front Immunol 14:1216548. 10.3389/fimmu.2023.1216548 37638049 10.3389/fimmu.2023.1216548 PMC10450946

[62] Li Z, Duan P, Qiu R, Fang L, Fang P, Xiao S (2023) HDAC6 degrades nsp8 of porcine deltacoronavirus through deacetylation and ubiquitination to inhibit viral replication. J Virol 97:e0037523. 10.1128/jvi.00375-23 37133375 10.1128/jvi.00375-23 PMC10231189

[63] Li Z, Xiao W, Yang Z, Guo J, Zhou J, Xiao S, Fang P, Fang L (2024) Cleavage of HDAC6 to dampen its antiviral activity by nsp5 is a common strategy of swine enteric coronaviruses. J Virol 98:e0181423. 10.1128/jvi.01814-23 38289103 10.1128/jvi.01814-23 PMC10878235

[64] Zhou Z, Sun Y, Yan X, Tang X, Li Q, Tan Y, Lan T, Ma J (2020) Swine acute diarrhea syndrome coronavirus (SADS-CoV) antagonizes interferon-β production via blocking IPS-1 and RIG-I. Virus Res 278:197843. 10.1016/j.virusres.2019.197843 31884203 10.1016/j.virusres.2019.197843 PMC7114844

[65] Zhong C, She G, Zhao Y, Liu Y, Li J, Wei X, Chen Z, Zhao K, Zhao Z, Xu Z, Zhang H, Cao Y, Xue C (2024) Swine acute diarrhea syndrome coronavirus Nsp1 suppresses IFN-λ1 production by degrading IRF1 via ubiquitin-proteasome pathway. Vet Res 55:45. 10.1186/s13567-024-01299-6 38589958 10.1186/s13567-024-01299-6 PMC11003034

[66] Duan Y, Yuan C, Suo X, Cao L, Kong X, Li X, Zheng H, Wang Q (2022) TET2 is required for Type I IFN-mediated inhibition of bat-origin swine acute diarrhea syndrome coronavirus. J Med Virol 94:3251–3256. 10.1002/jmv.27673 35211991 10.1002/jmv.27673

[67] Wang X, Qiu W, Hu G, Diao X, Li Y, Li Y, Li P, Liu Y, Feng Y, Xue C, Cao Y, Xu Z (2024) NS7a of SADS-CoV promotes viral infection via inducing apoptosis to suppress type III interferon production. J Virol 98:e0031724. 10.1128/jvi.00317-24 38624231 10.1128/jvi.00317-24 PMC11092342

[68] Zhang T, Yao J, Yang Z, Wang J, Yang K, Yao L (2024) Recombinant porcine interferon delta 8 inhibits swine acute diarrhoea syndrome coronavirus infection in vitro and in vivo. Vet Res 55:92. 10.1186/s13567-024-01346-2 39049059 10.1186/s13567-024-01346-2 PMC11270782

[69] Feng Y, Li X, Cassady K, Zou Z, Zhang X (2019) TET2 function in hematopoietic malignancies, immune regulation, and DNA repair. Front Oncol 9:210. 10.3389/fonc.2019.00210 31001476 10.3389/fonc.2019.00210 PMC6454012

[70] Cong B, Zhang Q, Cao X (2021) The function and regulation of TET2 in innate immunity and inflammation. Protein Cell 12:165–173. 10.1007/s13238-020-00796-6 33085059 10.1007/s13238-020-00796-6 PMC7895883

[71] Wang Q, Su L (2019) Vpr enhances HIV-1 Env processing and virion infectivity in macrophages by modulating TET2-dependent IFITM3 expression. MBio 10:e01344-19. 10.1128/mBio.01344-19 31431548 10.1128/mBio.01344-19 PMC6703422

[72] Ding Z, Fang L, Jing H, Zeng S, Wang D, Liu L, Zhang H, Luo R, Chen H, Xiao S (2014) Porcine epidemic diarrhea virus nucleocapsid protein antagonizes beta interferon production by sequestering the interaction between IRF3 and TBK1. J Virol 88:8936–8945. 10.1128/jvi.00700-14 24872591 10.1128/JVI.00700-14 PMC4136253

[73] Likai J, Shasha L, Wenxian Z, Jingjiao M, Jianhe S, Hengan W, Yaxian Y (2019) Porcine deltacoronavirus nucleocapsid protein suppressed IFN-β production by interfering porcine RIG-I dsRNA-binding and K63-linked polyubiquitination. Front Immunol 10:1024. 10.3389/fimmu.2019.01024 31143181 10.3389/fimmu.2019.01024 PMC6521028

[74] Liu Y, Liang QZ, Lu W, Le YY, Chen R, Huang YW, Wang B (2021) A comparative analysis of coronavirus nucleocapsid (N) proteins reveals the SADS-CoV N protein antagonizes IFN-β production by inducing ubiquitination of RIG-I. Front Immunol 12:688758. 10.3389/fimmu.2021.688758 34220846 10.3389/fimmu.2021.688758 PMC8242249

[75] Zhou Z, Sun Y, Xu J, Tang X, Zhou L, Li Q, Lan T, Ma J (2021) Swine acute diarrhea syndrome coronavirus nucleocapsid protein antagonizes interferon-β production via blocking the interaction between TRAF3 and TBK1. Front Immunol 12:573078. 10.3389/fimmu.2021.573078 33692778 10.3389/fimmu.2021.573078 PMC7937869

[76] Zhang J, Shi H, Zhang L, Feng T, Chen J, Zhang X, Ji Z, Jing Z, Zhu X, Liu D, Yang X, Zeng M, Shi D, Feng L (2024) Swine acute diarrhea syndrome coronavirus nucleocapsid protein antagonizes the IFN response through inhibiting TRIM25 oligomerization and functional activation of RIG-I/TRIM25. Vet Res 55:44. 10.1186/s13567-024-01303-z 38589930 10.1186/s13567-024-01303-z PMC11000385

[77] Van Gent M, Sparrer KMJ, Gack MU (2018) TRIM proteins and their roles in antiviral host defenses. Annu Rev Virol 5:385–405. 10.1146/annurev-virology-092917-043323 29949725 10.1146/annurev-virology-092917-043323 PMC6186430

[78] Zheng X, Wang X, Tu F, Wang Q, Fan Z, Gao G (2017) TRIM25 is required for the antiviral activity of zinc finger antiviral protein. J Virol 91:e00088-17. 10.1128/jvi.00088-17 28202764 10.1128/JVI.00088-17 PMC5391446

[79] Li MMH, Lau Z, Cheung P, Aguilar EG, Schneider WM, Bozzacco L, Molina H, Buehler E, Takaoka A, Rice CM, Felsenfeld DP, MacDonald MR (2017) TRIM25 enhances the antiviral action of zinc-finger antiviral protein (ZAP). PLoS Pathog 13:e1006145. 10.1371/journal.ppat.1006145 28060952 10.1371/journal.ppat.1006145 PMC5245905

[80] Meyerson NR, Zhou L, Guo YR, Zhao C, Tao YJ, Krug RM, Sawyer SL (2017) Nuclear TRIM25 specifically targets influenza virus ribonucleoproteins to block the onset of RNA chain elongation. Cell Host Microbe 22:627-638.e7. 10.1016/j.chom.2017.10.003 29107643 10.1016/j.chom.2017.10.003 PMC6309188

[81] Shen Z, Yang Y, Yang S, Zhang G, Xiao S, Fu ZF, Peng G (2020) Structural and biological basis of alphacoronavirus nsp1 associated with host proliferation and immune evasion. Viruses 12:812. 10.3390/v12080812 32731335 10.3390/v12080812 PMC7472224

[82] Xiang Y, Mou C, Shi K, Chen X, Meng X, Bao W, Chen Z (2023) SADS-CoV nsp1 inhibits the IFN-β production by preventing TBK1 phosphorylation and inducing CBP degradation. J Med Virol 95:e29104. 10.1002/jmv.29104 37721411 10.1002/jmv.29104

[83] Xiang Y, Mou C, Zhu L, Wang Z, Shi K, Bao W, Li J, Chen X, Chen Z (2024) SADS-CoV nsp1 inhibits the STAT1 phosphorylation by promoting K11/K48-linked polyubiquitination of JAK1 and blocks the STAT1 acetylation by degrading CBP. J Biol Chem 300:105779. 10.1016/j.jbc.2024.105779 38395305 10.1016/j.jbc.2024.105779 PMC10944115

[84] Huang HX, Zhao CC, Lei XX, Zhang XY, Li YY, Lan T, Zhao BP, Lu JY, Sun WC, Lu HJ, Jin NY (2023) Swine acute diarrhoea syndrome coronavirus (SADS-CoV) Nsp5 antagonizes type I interferon signaling by cleaving DCP1A. Front Immunol 14:1196031. 10.3389/fimmu.2023.1196031 37283741 10.3389/fimmu.2023.1196031 PMC10239798

[85] Tao R, Fang L, Bai D, Ke W, Zhou Y, Wang D, Xiao S (2018) Porcine reproductive and respiratory syndrome virus nonstructural protein 4 cleaves porcine DCP1a to attenuate its antiviral activity. J Immunol 201:2345–2353. 10.4049/jimmunol.1701773 30158128 10.4049/jimmunol.1701773

[86] Zhu X, Chen J, Tian L, Zhou Y, Xu S, Long S, Wang D, Fang L, Xiao S (2020) Porcine deltacoronavirus nsp5 cleaves DCP1A to decrease its antiviral activity. J Virol 94:e02162-19. 10.1128/JVI.02162-19 32461317 10.1128/JVI.02162-19 PMC7375372

[87] Song L, Wang D, Abbas G, Li M, Cui M, Wang J, Lin Z, Zhang XE (2023) The main protease of SARS-CoV-2 cleaves histone deacetylases and DCP1A, attenuating the immune defense of the interferon-stimulated genes. J Biol Chem 299:102990. 10.1016/j.jbc.2023.102990 36758802 10.1016/j.jbc.2023.102990 PMC9907797

[88] Sommereyns C, Paul S, Staeheli P, Michiels T (2008) IFN-lambda (IFN-λ) is expressed in a tissue-dependent fashion and primarily acts on epithelial cells in vivo. PLoS Pathog 4:e1000017. 10.1371/journal.ppat.1000017 18369468 10.1371/journal.ppat.1000017 PMC2265414

[89] Mahlakõiv T, Hernandez P, Gronke K, Diefenbach A, Staeheli P (2015) Leukocyte-derived IFN-α/β and epithelial IFN-λ constitute a compartmentalized mucosal defense system that restricts enteric virus infections. PLoS Pathog 11:e1004782. 10.1371/journal.ppat.1004782 25849543 10.1371/journal.ppat.1004782 PMC4388470

[90] Chen T, Tu S, Ding L, Jin M, Chen H, Zhou H (2023) The role of autophagy in viral infections. J Biomed Sci 30:5. 10.1186/s12929-023-00899-2 36653801 10.1186/s12929-023-00899-2 PMC9846652

[91] Yamamoto H, Zhang S, Mizushima N (2023) Autophagy genes in biology and disease. Nat Rev Genet 24:382–400. 10.1038/s41576-022-00562-w 36635405 10.1038/s41576-022-00562-w PMC9838376

[92] Choi Y, Bowman JW, Jung JU (2018) Autophagy during viral infection—a double-edged sword. Nat Rev Microbiol 16:341–354. 10.1038/s41579-018-0003-6 29556036 10.1038/s41579-018-0003-6 PMC6907743

[93] Chawla K, Subramanian G, Rahman T, Fan S, Chakravarty S, Gujja S, Demchak H, Chakravarti R, Chattopadhyay S (2022) Autophagy in virus infection: a race between host immune response and viral antagonism. Immuno 2:153–169. 10.3390/immuno2010012 35252965 10.3390/immuno2010012 PMC8893043

[94] Matsuzawa-Ishimoto Y, Hwang S, Cadwell K (2018) Autophagy and inflammation. Annu Rev Immunol 36:73–101. 10.1146/annurev-immunol-042617-053253 29144836 10.1146/annurev-immunol-042617-053253

[95] Sagnier S, Daussy CF, Borel S, Robert-Hebmann V, Faure M, Blanchet FP, Beaumelle B, Biard-Piechaczyk M, Espert L (2015) Autophagy restricts HIV-1 infection by selectively degrading Tat in CD4+ T lymphocytes. J Virol 89:615–625. 10.1128/jvi.02174-14 25339774 10.1128/JVI.02174-14 PMC4301118

[96] Liu Y, Gordesky-Gold B, Leney-Greene M, Weinbren NL, Tudor M, Cherry S (2018) Inflammation-induced, STING-dependent autophagy restricts Zika virus infection in the *Drosophila* brain. Cell Host Microbe 24:57-68.e3. 10.1016/j.chom.2018.05.022 29934091 10.1016/j.chom.2018.05.022 PMC6173519

[97] Delorme-Axford E, Klionsky DJ (2019) Inflammatory-dependent Sting activation induces antiviral autophagy to limit zika virus in the *Drosophila* brain. Autophagy 15:1–3. 10.1080/15548627.2018.1539585 30354937 10.1080/15548627.2018.1539585 PMC6287683

[98] Kim N, Kim MJ, Sung PS, Bae YC, Shin EC, Yoo JY (2016) Interferon-inducible protein SCOTIN interferes with HCV replication through the autolysosomal degradation of NS5A. Nat Commun 7:10631. 10.1038/ncomms10631 26868272 10.1038/ncomms10631 PMC4754343

[99] Biering SB, Choi J, Halstrom RA, Brown HM, Beatty WL, Lee S, McCune BT, Dominici E, Williams LE, Orchard RC, Wilen CB, Yamamoto M, Coers J, Taylor GA, Hwang S (2017) Viral replication complexes are targeted by LC3-guided interferon-inducible GTPases. Cell Host Microbe 22:74-85.e7. 10.1016/j.chom.2017.06.005 28669671 10.1016/j.chom.2017.06.005 PMC5591033

[100] Richetta C, Grégoire IP, Verlhac P, Azocar O, Baguet J, Flacher M, Tangy F, Rabourdin-Combe C, Faure M (2013) Sustained autophagy contributes to measles virus infectivity. PLoS Pathog 9:e1003599. 10.1371/journal.ppat.1003599 24086130 10.1371/journal.ppat.1003599 PMC3784470

[101] Buckingham EM, Carpenter JE, Jackson W, Grose C (2014) Autophagy and the effects of its inhibition on varicella-zoster virus glycoprotein biosynthesis and infectivity. J Virol 88:890–902. 10.1128/jvi.02646-13 24198400 10.1128/JVI.02646-13 PMC3911683

[102] Beale R, Wise H, Stuart A, Ravenhill BJ, Digard P, Randow F (2014) A LC3-interacting motif in the influenza A virus M2 protein is required to subvert autophagy and maintain virion stability. Cell Host Microbe 15:239–247. 10.1016/j.chom.2014.01.006 24528869 10.1016/j.chom.2014.01.006 PMC3991421

[103] Yakoub AM, Shukla D (2015) Basal autophagy is required for herpes simplex virus-2 infection. Sci Rep 5:12985. 10.1038/srep12985 26248741 10.1038/srep12985 PMC4528227

[104] Li M, Li J, Zeng R, Yang J, Liu J, Zhang Z, Song X, Yao Z, Ma C, Li W, Wang K, Wei L (2018) Respiratory syncytial virus replication is promoted by autophagy-mediated inhibition of apoptosis. J Virol 92:e02193-17. 10.1128/JVI.02193-17 29386287 10.1128/JVI.02193-17 PMC5874425

[105] Wang R, Zhu Y, Zhao J, Ren C, Li P, Chen H, Jin M, Zhou H (2019) Autophagy promotes replication of influenza A virus in vitro. J Virol 93:e01984-18. 10.1128/jvi.01984-18 30541828 10.1128/JVI.01984-18 PMC6363991

[106] Li M, Guo L, Feng L (2022) Interplay between swine enteric coronaviruses and host innate immune. Front Vet Sci 9:1083605. 10.3389/fvets.2022.1083605 36619958 10.3389/fvets.2022.1083605 PMC9814124

[107] Yan Q, Liu X, Sun Y, Zeng W, Li Y, Zhao F, Wu K, Fan S, Zhao M, Chen J, Yi L (2022) Swine enteric coronavirus: diverse pathogen–host interactions. Int J Mol Sci 23:3953. 10.3390/ijms23073953 35409315 10.3390/ijms23073953 PMC8999375

[108] Chen YM, Burrough E (2022) The effects of swine coronaviruses on ER stress, autophagy, apoptosis, and alterations in cell morphology. Pathogens 11:940. 10.3390/pathogens11080940 36015060 10.3390/pathogens11080940 PMC9416022

[109] Zeng S, Zhao Y, Peng O, Xia Y, Xu Q, Li H, Xue C, Cao Y, Zhang H (2022) Swine acute diarrhea syndrome coronavirus induces autophagy to promote its replication via the Akt/mTOR pathway. iScience 25:105394. 10.1016/j.isci.2022.105394 36281226 10.1016/j.isci.2022.105394 PMC9581643

[110] Shi D, Zhou L, Shi H, Zhang J, Zhang J, Zhang L, Liu D, Feng T, Zeng M, Chen J, Zhang X, Xue M, Jing Z, Liu J, Ji Z, He H, Guo L, Wu Y, Ma J et al (2023) Autophagy is induced by swine acute diarrhea syndrome coronavirus through the cellular IRE1-JNK-Beclin 1 signaling pathway after an interaction of viral membrane-associated papain-like protease and GRP78. PLoS Pathog 19:e1011201. 10.1371/journal.ppat.1011201 36888569 10.1371/journal.ppat.1011201 PMC9994726

[111] Kerr JFR, Wyllie AH, Currie AR (1972) Apoptosis: a basic biological phenomenon with wide-ranging implications in tissue kinetics. Br J Cancer 26:239–257. 10.1038/bjc.1972.33 4561027 10.1038/bjc.1972.33 PMC2008650

[112] Nössing C, Ryan KM (2023) 50 years on and still very much alive: ‘apoptosis: a basic biological phenomenon with wide-ranging implications in tissue kinetics.’ Br J Cancer 128:426–431. 10.1038/s41416-022-02020-0 36369364 10.1038/s41416-022-02020-0 PMC9938139

[113] Bertheloot D, Latz E, Franklin BS (2021) Necroptosis, pyroptosis and apoptosis: an intricate game of cell death. Cell Mol Immunol 18:1106–1121. 10.1038/s41423-020-00630-3 33785842 10.1038/s41423-020-00630-3 PMC8008022

[114] Ketelut-Carneiro N, Fitzgerald KA (2022) Apoptosis, pyroptosis, and necroptosis—oh my! The many ways a cell can die. J Mol Biol 434:167378. 10.1016/j.jmb.2021.167378 34838807 10.1016/j.jmb.2021.167378

[115] Chattopadhyay S, Yamashita M, Zhang Y, Sen GC (2011) The IRF-3/Bax-mediated apoptotic pathway, activated by viral cytoplasmic RNA and DNA, inhibits virus replication. J Virol 85:3708–3716. 10.1128/jvi.02133-10 21307205 10.1128/JVI.02133-10 PMC3126131

[116] Chattopadhyay S, Kuzmanovic T, Zhang Y, Wetzel JL, Sen GC (2016) Ubiquitination of the transcription factor IRF-3 activates RIPA, the apoptotic pathway that protects mice from viral pathogenesis. Immunity 44:1151–1161. 10.1016/j.immuni.2016.04.009 27178468 10.1016/j.immuni.2016.04.009 PMC4991351

[117] Everett H, McFadden G (1999) Apoptosis: an innate immune response to virus infection. Trends Microbiol 7:160–165. 10.1016/S0966-842X(99)01487-0 10217831 10.1016/s0966-842x(99)01487-0

[118] Orzalli MH, Kagan JC (2017) Apoptosis and necroptosis as host defense strategies to prevent viral infection. Trends Cell Biol 27:800–809. 10.1016/j.tcb.2017.05.007 28642032 10.1016/j.tcb.2017.05.007 PMC5653411

[119] Xu Z, Zhang Y, Cao Y (2020) The roles of apoptosis in swine response to viral infection and pathogenesis of swine enteropathogenic coronaviruses. Front Vet Sci 7:572425. 10.3389/fvets.2020.572425 33324698 10.3389/fvets.2020.572425 PMC7725767

[120] Amara A, Mercer J (2015) Viral apoptotic mimicry. Nat Rev Microbiol 13:461–469. 10.1038/nrmicro3469 26052667 10.1038/nrmicro3469 PMC7097103

[121] Zhou X, Jiang W, Liu Z, Liu S, Liang X (2017) Virus infection and death receptor-mediated apoptosis. Viruses 9:316. 10.3390/v9110316 29077026 10.3390/v9110316 PMC5707523

[122] Naderer T, Fulcher MC (2018) Targeting apoptosis pathways in infections. J Leukoc Biol 103:275–285. 10.1189/jlb.4mr0717-286r 29372933 10.1189/JLB.4MR0717-286R

[123] Zhang J, Han Y, Shi H, Chen J, Zhang X, Wang X, Zhou L, Liu J, Zhang J, Ji Z, Jing Z, Ma J, Shi D, Feng L (2020) Swine acute diarrhea syndrome coronavirus-induced apoptosis is caspase- and cyclophilin D-dependent. Emerg Microbes Infect 9:439–456. 10.1080/22221751.2020.1722758 32090691 10.1080/22221751.2020.1722758 PMC7054944

[124] Zhang J, Zhang L, Shi H, Feng S, Feng T, Chen J, Zhang X, Han Y, Liu J, Wang Y, Ji Z, Jing Z, Liu D, Shi D, Feng L (2022) Swine acute diarrhea syndrome coronavirus replication is reduced by inhibition of the extracellular signal-regulated kinase (ERK) signaling pathway. Virology 565:96–105. 10.1016/j.virol.2021.10.009 34768113 10.1016/j.virol.2021.10.009 PMC8556614

